# Does the Brush-Sign Reflect Collateral Status and DWI-ASPECTS in Large Vessel Occlusion?

**DOI:** 10.3389/fneur.2022.828256

**Published:** 2022-03-02

**Authors:** Lucie Rascle, Alexandre Bani Sadr, Camille Amaz, Nathan Mewton, Marielle Buisson, Marc Hermier, Elodie Ong, Julia Fontaine, Laurent Derex, Yves Berthezène, Omer Faruk Eker, Tae-Hee Cho, Norbert Nighoghossian, Laura Mechtouff

**Affiliations:** ^1^Department of Stroke, Hospices Civils de Lyon, Lyon, France; ^2^Department of Neuroradiology, Hospices Civils de Lyon, Lyon, France; ^3^Clinical Investigation Center, Hospices Civils de Lyon, INSERM, Lyon, France; ^4^CarMeN Laboratory, INSERM, University Lyon 1, Lyon, France

**Keywords:** brush-sign, collaterality, lesion volume, thrombectomy, MRI, DWI-ASPECTS

## Abstract

**Introduction:**

The relevance of the brush-sign remained poorly documented in large vessel occlusion (LVO). We aimed to assess the relationship between the brush-sign and collateral status and its potential impact on baseline diffusion-weighted imaging–Alberta Stroke Program Early Computed Tomography Score (DWI-ASPECTS) in acute ischemic stroke (AIS) patients eligible to mechanical thrombectomy (MT).

**Methods:**

Consecutive patients admitted in the Lyon Stroke Center with anterior circulation AIS due to intracranial internal carotid artery (ICA) and/or M1 or M2 segment of the middle cerebral artery (MCA) occlusion eligible for MT were included. The brush-sign was assessed on T2-gradient-echo MRI. Collateral status was assessed on digital subtraction angiography according to the American Society of Interventional and Therapeutic Neuroradiology/Society of Interventional Radiology (ASITN/SIR) score.

**Results:**

In this study, 504 patients were included, among which 171 (33.9%) patients had a brush-sign. Patients with a brush-sign more frequently had a poor collateral status [72 (42.1%) vs. 103 (30.9%); *p* = 0.017]. In univariable analysis, a DWI-ASPECTS < 7 was associated with a brush sign. Following multivariable analysis, the brush-sign no longer affected DWI-ASPECTS < 7 while the latter remained associated with younger age [odds ratio (OR) 0.97, 95% CI.96–0.99], male sex (OR 1.79, 95% CI 1.08–2.99), a higher National Institutes of Health Stroke Scale (NIHSS) score (OR 1.16, 95% CI 1.1–1.21), a poor collateral status (OR 9.35, 95% CI 5.59-16.02), MCA segment (OR 2.54, 95% CI 1.25–5.38), and intracranial ICA (OR 3.01, 95% CI 1.16–8) occlusion.

**Conclusions and Relevance:**

The brush-sign may be a marker of poor collateral status but did not independently predict a lower DWI-ASPECTS.

**Clinical Trial Registration:**

ClinicalTrials.gov, identifier: NCT04620642.

## Introduction

Assessment of vessels using gradient recalled echo T2^*^-weighted imaging (GRE T2^*^WI) may provide insights into acute ischemic stroke (AIS) pathophysiology (1–4). The brush-sign refers to the abnormal visibility of enlarged deep medullary veins on GRE T2^*^WI related to an increase of blood deoxyhemoglobin concentration (3–6). The brush-sign has aroused significant interest in recent years. Previous studies have reported that the brush-sign was a marker of severe ischemia and hemorrhagic transformation risk in the context of intravenous (IV) thrombolytic therapy, while others have highlighted its relationship with a poor prognosis (5, 7–9). The clinical relevance of brush-sign in large vessel occlusion (LVO), and especially its link to the collateral status and baseline lesion volume assessed with diffusion-weighted imaging–Alberta Stroke Program Early Computed Tomography Score (DWI-ASPECTS) remained poorly documented in the context of mechanical thrombectomy (MT). We evaluated this relationship through a large registry of patients with AIS along with LVO eligible for MT.

## Materials and Methods

### Standard Protocol Approvals, Registrations, and Patient Consents

The study was approved by the local ethics committee and the French Data Protection Authority. All participants have given their agreement to the use of their medical data.

### Study Population

The Lyon Registry of Stroke Treated by Thrombolysis or Thrombectomy (RELATE) (NCT NCT04620642) collected data from consecutive patients admitted in the Lyon Stroke Center for AIS eligible for MT and/or IV thrombolysis. For the present study, only patients with anterior circulation AIS with occlusion of the intracranial internal carotid artery (ICA) and/or M1 or M2 segment of the middle cerebral artery (MCA) on admission brain magnetic resonance imaging (MRI) and eligible for MT from January 2015 to October 2020 were considered. Patients treated with IV thrombolysis alone were not included in the present study. Demographic characteristics and medical history as well as the National Institute of Health Stroke Scale (NIHSS) score assessed by board-certified neurologists were collected at admission.

### Neuroimaging Protocol

Brain MRIs were obtained with the 1.5 or 3 T Ingenia scanners (Philips Healthcare, Best, The Netherlands). Admission brain MRI protocol included diffusion-weighted-imaging (DWI), GRE T2^*^WI, fluid-attenuated inversion recovery (FLAIR), and 3D time-of-flight (TOF) MR angiography.

### Image Analysis

**Figure 1:**
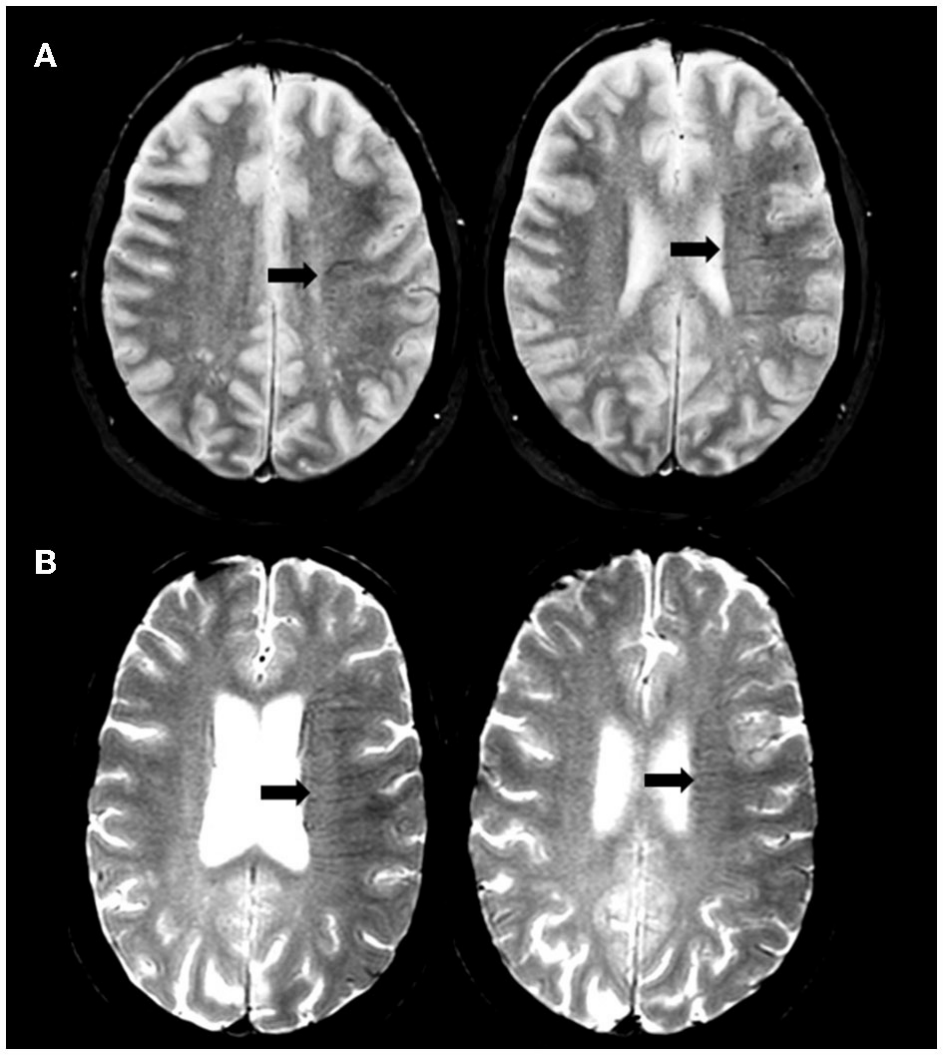
Illustration of brush-sign. Axial gradient-recalled echo T2*-weighted imaging showing moderate **(A)** and obvious **(B)** brush-sign.

Two experienced physicians in stroke imaging (LR and NN) blinded to clinical and digital angiography data independently reviewed MRI. The brush-sign was defined as multiple hypointense linear and/or branching structures extending through the affected hemisphere, parallel or perpendicular to the outer wall of the lateral ventricles along the course of deep medullary veins on the baseline T2^*^-weighted gradient-echo sequence (10). The brush-sign was categorized as absent or present and if present classified as moderate, or obvious (Figure 1) (5). Any discrepancies were resolved by a third expert (ABS). DWI-ASPECTS was assessed on admission DWI sequence without further details on the affected brain regions (11). White matter hyperintensities (WMHs) were assessed on the FLAIR sequence according to the Fazekas scale; both the periventricular and subcortical components of the scale were evaluated (12). Cerebral microbleeds (CMB) were rated on GRE T2^*^WI according to the MARS criteria (13). The collateral status was determined on digital subtraction angiography (DSA) images according to the score of the American Society of Interventional and Therapeutic Neuroradiology/Society of Interventional Radiology (ASITN/SIR) and considered as poor for ASITN/SIR score < 3 (14). Reperfusion status was considered as successful if Thrombolysis in Cerebral Infarction (TICI) score was 2b or 3 (14). A CT scan was performed on day 1. Symptomatic intracerebral hemorrhage (sICH) was defined according to the SITS-MOST definition (15).

### Statistical Analysis

Continuous variables are expressed as means (*SD*) or medians (IQR) depending on their distributions, and categorical variables as percentages. Medians were compared using the Mann-Whitney test. Percentages were compared using Fisher's exact test. Interrater agreement for the evaluation of the brush-sign was assessed using the Cohen's kappa coefficient. DWI-ASPECTS was dichotomized at 7, which is a reliable surrogate of DWI volume of 100 ml (16). Univariable and multivariable logistic regression (forward method) models were used to determine the factors associated with DWI-ASPECTS < 7. The same analysis was performed in the subset of patients with M1 MCA segment occlusion. Covariates identified as statistically significant in univariable analyses (*p* < 0.05) were implemented through a backward procedure with Akaike Information Criterion (AIC) minimization. The interaction between the brush-sign and collateral status to predict DWI-ASPECTS was tested. Statistical testing used a two-tailed α level of 0.05. The data were analyzed with R statistical software version 3.6.1.

### Data Availability Statement

Further anonymized data can be made available to qualified investigators on request to the corresponding author.

## Results

### Study Population

From January 2015 to October 2020, 1,434 patients with anterior circulation AIS with LVO were eligible for MT in the Lyon Stroke Center. Among them, 305 with a CT scan as first-line imaging were excluded. Of the remaining 1,129 patients, 160 patients had uninterpretable MRI data and 465 had non-assessable ASITN/SIR score (anterograde flow, internal carotid artery (ICA) terminus occlusion, and inadequate acquisition) and were therefore excluded (Supplementary Figure 1). These patients did not differ from included patients in terms of age, sex and time from stroke onset to imaging but had a higher admission NIHSS score (17 [12**–**21] vs. 15 [9–19]; *p* < 0.001), a lower DWI-ASPECTS (7 [6–8] vs. 7 [6–8]; *p* < 0.001), a lower rate of M2 [57 (9.1%) vs. 100 (19.8%)] and M1 MCA segments [341 (54.6%) vs. 349 (69.2%)] occlusion and a higher rate of intracranial ICA [215 (34.4%) vs. 55 (10.9%); *p* for all intracranial occlusion site < 0.001] as well as tandem [155 (24.8%) vs. 45 (7.2%); *p* <0.001] occlusion compared with included patients. The remaining 504 patients represent the study population. Mean age was 69.4 +/−16 years, 235 (46.6%) were men. Median NIHSS on admission was 15 [9–19] and median DWI-ASPECTS was 7 [6–8]. The median delay from stroke onset to imaging was 106 min [81–153].

### Prevalence and Distribution of the Brush-Sign

**Figure 2:**
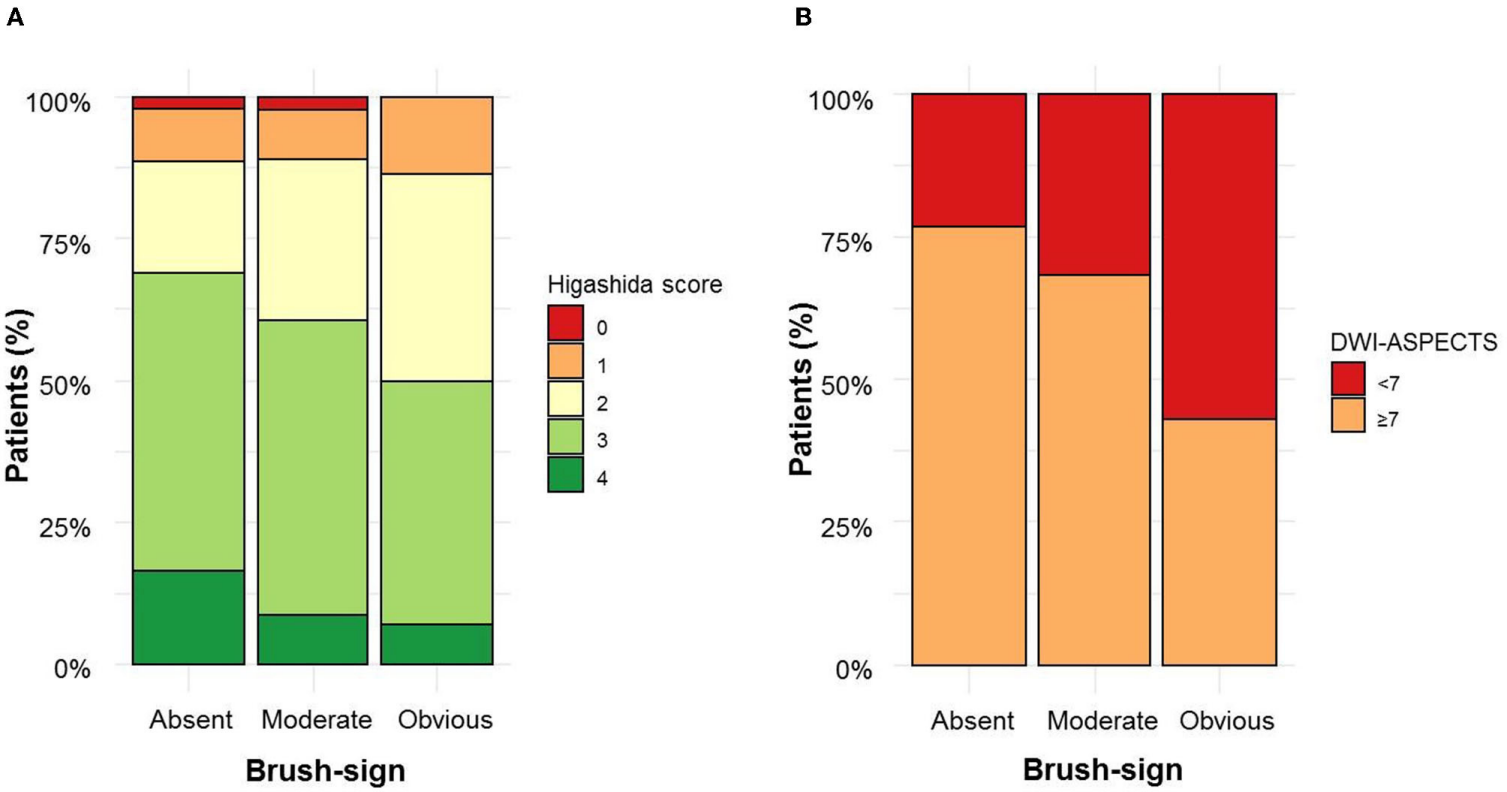
Brush sign severity according to collateral status **(A)** and diffusion-weighted imaging–Alberta Stroke Program Early Computed Tomography Score (DWI-ASPECTS) **(B)**.

A total of 171 (33.9%) patients had a brush-sign [moderate, *n* = 127 (25.2%); obvious, *n* = 44 (8.7%)]. In the subset of patients with M1 MCA segment occlusion (*n* = 349), 116 (33,2%) had a brush-sign. Interrater agreement for the evaluation of brush-sign presence was substantial (kappa =0.74). Adjudication was needed for 50 (10%) patients. The main characteristics of the study population are shown in Table 1 according to the brush-sign presence. Patients with a brush-sign were younger, more often male, had a higher NIHSS score, a lower DWI-ASPECTS, a poor collateral status, and received IV thrombolysis more often afterward. The distribution of collateral status and DWI-ASPECTS according to the brush-sign severity are illustrated in Figure 2. The brush-sign was not associated with the severity of WMH, the presence of CMB, intracranial occlusion site, reperfusion status, or the occurrence of sICH. A brush sign was detected in 28% (117/418) and 63% (54/86) of patients who underwent a 1.5T and a 3T MRI, respectively.

**Table 1:** Main clinical and imaging characteristics according to the presence of the brush-sign.

	**Brush-sign presence (*n* = 171)**	**Brush-sign absence (*n* = 333)**	***p*-value**
Age, years	66.3 +/– 16.0	70.9 +/– 15.8	**0.002**
Male	102 (59.6)	133 (39.9)	**<0.001**
NIHSS score	16 [11–20]	13 [8–18]	**<0.001**
Wake-up stroke^a^	48 (28.2)	94 (28.3)	1.000
Magnetic field strength			**<0.001**
1.5T MRI	117 (68.4)	301 (90.4)	
3T MRI	54 (31.6)	32 (9.6)	
DWI-ASPECTS	7 [6–8]	8 [7–8]	**<0.001**
Fazekas' score ≥3^b^	46 (34.6)	106 (37.5)	0.647
Cerebral microbleeds ≥1^b^	15 (11.3)	53 (18.8)	0.074
Poor collateral status	72 (42.1)	103 (30.9)	**0.017**
Intracranial occlusion site			0.413
M2 MCA segment	32 (18.7)	68 (20.4)	
M1 MCA segment	116 (67.8)	233 (70.0)	
Intracranial ICA^c^	23 (13.5)	32 (9.6)	
Tandem occlusion	17 (9.9)	28 (8.4)	0.684
IV thrombolysis	112 (65.5)	169 (50.8)	**0.002**
Successful reperfusion	123 (71.9)	243 (73.0)	0.886
Stroke onset to imaging, min^a^	105 [81–137]	108 [81–167]	0.133
sICH^d^	4 (2.8)	3 (1.1)	0.380

*NIHSS, national institute of health stroke score; MCA, middle cerebral artery; ICA, internal carotid artery; DWI-ASPECTS, diffusion-weighted imaging-Alberta stroke program early CT score; IV, intravenous; sICH, symptomatic intracerebral hemorrhage*.

*Variables are displayed as absolute number (percentage of column total), mean ± SD, or median (25th−75th percentiles) as appropriate*.

*Bold values indicate p < 0.05*.

a *Ten patients missing data*.

b *Eighty-eight patients missing data*.

c *Intracranial ICA occlusion was associated with M1 and M2 MCA segment occlusion in 53 and two patients, respectively*.

d *Ninety-two patients missing data*.

### Factors Associated With DWI-ASPECTS in the Whole Study Population

On univariable analysis, a DWI-ASPECTS < 7 was associated with younger age, male sex, a higher NIHSS score, a poor collateral status, a brush-sign, M1 MCA segment, intracranial ICA, and tandem occlusion. Following multivariable analysis, after adjustment for age, sex, NIHSS score, intracranial occlusion site, tandem occlusion, and collateral status, the brush-sign was no longer associated with a DWI-ASPECTS < 7. Only a younger age, male sex, a higher NIHSS score, a poor collateral status, M1 MCA segment, and intracranial ICA occlusion remained associated with a DWI-ASPECTS < 7 (Table 2). We did not find any interaction between collateral status and the brush-sign, which means that the brush-sign presence did not affect the relationship between collateral status and DWI-ASPECTS as illustrated in Supplementary Figure 2.

**Table 2:** Factors associated with diffusion-weighted imaging–Alberta Stroke Program Early Computed Tomography Score (DWI-ASPECTS) < 7 in univariable and multivariable analysis.

	**Crude OR (95% CI)**	***p*-value**	**Adjusted OR (95% CI)^a^**	***p*-value**
Age	0.98 (0.97–0.99)	<0.01	0.97 (0.96–0.99)	<0.01
Male	2.27 (1.53–3.39)	<0.01	1.79 (1.08–2.99)	0.02
NIHSS score	1.15 (1.11–1.20)	<0.01	1.16 (1.10–1.21)	<0.01
Brush-sign	2.05 (1.37–3.06)	<0.01	-	-
Poor collateral status	9.05 (5.86–14.19)	<0.01	9.35 (5.59–16.02)	<0.01
M1 MCA segment occlusion^b^	1.81 (1.05–3.26)	0.04	2.54 (1.25–5.38)	0.01
Intracranial ICA occlusion^b^	3.93 (1.89–8.34)	<0.01	3.01 (1.16–8.00)	0.02
Tandem occlusion	2.43 (1.30–4.53)	0.01	-	-

*OR, odds ratio; NIHSS, national institute of health stroke score; MCA, middle cerebral artery; ICA, internal carotid artery*.

a *Model was adjusted for age, sex, NIHSS score, collateral status, and intracranial occlusion site (brush-sign and tandem occlusion not retained by the backward selection)*.

b *vs. M2 middle cerebral artery segment occlusion*.

### Factors Associated With DWI-ASPECTS in Patients With M1 MCA Segment Occlusion

Among the 349 patients with M1 MCA segment occlusion, the univariable analysis showed that DWI-ASPECTS < 7 was associated with male sex, a higher NIHSS score, a brush-sign, a poor collateral status (Supplementary Table 1). On multivariable analysis, only male sex, a higher NIHSS score, and a poor collateral status remained associated with a DWI-ASPECTS < 7. Nor did we find any interaction between collateral status and the brush-sign to predict a DWI-ASPECTS < 7.

## Discussion

Our study conducted on a large sample of patients with AIS along with LVO eligible for MT showed that the brush-sign was a marker of collateral status but did not predict independently a lower baseline DWI-ASPECTS.

In AIS, the increase of oxygen extraction fraction needed to maintain a constant cerebral metabolic rate of oxygen (CMRO2) leads to an increased level of deoxyhemoglobin within deep medullary veins (17, 18). This results in a signal drop on GRE T2^*^WI due to the paramagnetic properties of deoxyhemoglobin (3, 4). In addition, the dilatation of cerebral veins and brain acidosis may contribute to signal changes (19). Up to now, the relationship between the brush-sign and collateral status has not been addressed. We elaborated the hypothesis that a decreased collaterality supply may promote a low flow state leading to increased deoxygenation within the venous system.

Existing pieces of evidence on the relationship between veins' abnormal visibility and collateral status are often not focused on deep medullary veins analysis and offer divergent results (20–23). Xu et al. found a relationship between the presence of asymmetric deep medullary veins and a good leptomeningeal collateral circulation among 56 patients with AIS along with LVO (23). Park et al. showed an association between extensive prominent vessel signs (cortical and/or deep medullary vein) and a good collateral flow in 80 patients with LVO (20). However, in these two studies, assessment of collateral status using FLAIR imaging +/– post-contrast TOF MR angiography appears as a limitation (24). In addition, in the second study, MRI was performed within 3 days from stroke onset. Thus this delay may have concealed retrograde (or anterograde) flow due to extensive infarction and related edema (25). In contrast, Verma et al. observed that prominent cortical veins grade was associated with a poor leptomeningeal collateral status on DSA among 33 patients with acute occlusion of the M1 MCA segment (21). And a recent study conducted in 152 patients with AIS along with LVO found a correlation between prominent vessel sign (cortical and/or deep medullary vein) score and collateral status grade defined on the multiphase MR angiography collateral map (22). Focusing on deep medullary veins in a large sample of patients with AIS along with LVO, we found that brush-sign was associated with a poor collateral status assessed on pretreatment DSA. These latter observations suggest that the collateral status decides the ratio of deoxyhemoglobin–oxyhemoglobin in the venous system.

As the collateral status and hemodynamic impairment are closely linked to the baseline lesion volume, we further explored the relationship between brush-sign and DWI-ASPECTS. Guenego et al. have observed that the brush-sign may be uncoupled from collateral status but interacted with this latter to predict core infarct volume (26). Under favorable collateral blood-flow, patients with a brush-sign had the lowest core infarct volume. In contrast, under poor collateral blood-flow, the brush-sign was associated with the highest core infarct volume. In our study, the brush-sign did not appear as an independent predictor of a low DWI-ASPECTS in multivariable analysis, neither in the whole study population nor in the subset of patients with M1 MCA segment occlusion. Nor did we not observe any interaction between the brush-sign and collateral status to predict DWI-ASPECTS.

In the present study, about 1/3 of patients had a brush-sign according to the double reading of MRI. This frequency seems lower than those reported in most of the previous studies. Indeed, other authors, except Wang, et al. have reported a frequency of the brush-sign ranging from 47 to 96% (5–9, 20, 22, 23, 26). Several factors may explain this heterogeneity, such as the variable inclusion criteria (intracranial occlusion site, delay from stroke onset to MRI), the use of different sequences [T2-gradient-echo or susceptibility-weighted imaging (SWI)], or magnetic fields (1.5T or 3T MRI), as well as a combined analysis of deep medullary and cortical veins in some studies.

The strength of our study lies in the large sample of patients, allowing a robust analysis of the predictors of core infarct volume. In addition, we assessed collateral status with DSA that is considered as the “gold standard”. However, this method faces technical limits in patients with intracranial ACI occlusion. Among them, only patients with the persistence of residual anterograde flow or the use of contralateral catheterization had data about collateral status, thus leading to exclusion of a substantial proportion of patients from the analysis (14).

We acknowledge some limitations besides the monocentric and retrospective design. Merging patients with occlusion of intracranial ICA and/or M1 or M2 segment of the MCA may generate a certain level of heterogeneity although it has been widely used in previous studies (5–9, 20, 22, 23). However, subgroup analysis of patients with M1 MCA occlusion did not significantly modify the results and did not alter the interpretation of the data. Next, the sensitivity of the GRE T2^*^ sequence is lower than SWI for the detection of the brush-sign (27). SWI, as a high-spatial-resolution 3-dimensional gradient-echo MR technique, is highly susceptible to paramagnetic substances and has demonstrated advantages over conventional GRE T2^*^WI (28, 29). Although delays and methods of image acquisition have improved, this method was previously considered time-consuming in an emergency setting. Furthermore, as the brush-sign was assessed through a qualitative approach, there may be bias in the interpretation of images. Quantitative susceptibility mapping is a development of SWI that can visualize veins and quantify blood oxygen saturation by measuring susceptibility values. This method may accurately identify hypointense vessels and provide more quantitative information about cerebral ischemia (30, 31). In addition, although DWI-ASPECTS is increasingly used in patients with AIS, this semi-quantitative analysis is less precise than a quantitative assessment of lesion volumes. It may overlook lesions within the striatocapsular region and only partially covers the MCA territory. This explains the wide range of true lesion volumes for a given DWI-ASPECTS (16). However, DWI-ASPECTS cut-off of 7 may be used as a reliable surrogate of DWI volume cut-off of 70 mL (16). Last, the use of either 1.5 T or 3 T magnetic fields related to MRI magnets availability may affect the frequency of the brush-sign (32). As 1.5T MRI has been performed in about 80% of the study population, our results can only be applied when 1.5 T MRI is used.

## Summary/conclusions

In this large sample of patients with AIS along with LVO eligible for MT, the brush-sign appears as a marker of poor collateral status while it did not independently predict a low DWI-ASPECTS. Factors associated with the brush-sign as well as the prognostic value of the latter, not assessed here, deserve further exploration.

## Data Availability Statement

The raw data supporting the conclusions of this article will be made available by the authors, without undue reservation.

## Ethics Statement

The studies involving human participants were reviewed and approved by Local Ethic Committee. Written informed consent for participation was not required for this study in accordance with the national legislation and the institutionalrequirements.

## Author Contributions

LR, LM, NN, and CA designed the study, had a major role in the acquisition and interpretation of data, and drafted the manuscript for intellectual content. AB, MB, EO, JF, LD, and YB had a major role in the acquisition of data and in the revision of the manuscript for intellectual content. T-HC, OE, NM, and MH designed the study and had a major role in the acquisition of data and in the revision of the manuscript for intellectual content. CA, NM, and MB provide statistical analysis and improved methodology. All authors contributed to the article and approved the submitted version.

## Funding

This work was supported by the RHU MARVELOUS (ANR-16-RHUS-0009) of Université de Lyon, within the program Investissements d'Avenir operated by the French National Research Agency.

## Conflict of Interest

The authors declare that the research was conducted in the absence of any commercial or financial relationships that could be construed as a potential conflict of interest.

## Publisher's Note

All claims expressed in this article are solely those of the authors and do not necessarily represent those of their affiliated organizations, or those of the publisher, the editors and the reviewers. Any product that may be evaluated in this article, or claim that may be made by its manufacturer, is not guaranteed or endorsed by the publisher.
