# Supplementary material for: Does the Brush-Sign Reflect Collateral Status and DWI-ASPECTS in Large Vessel Occlusion?
Source: Front Neurol. 2022 Mar 2;13:828256. doi: 10.3389/fneur.2022.828256 (PMC8924293; doi:10.3389/fneur.2022.828256)
Supplement: Supplementary file 1 [file Data_Sheet_1.PDF]

Supplementary Figure 1. Study Flow Chart

Supplementary Figure 2. Relationship between diffusion-weighted imaging–Alberta Stroke Program Early Computed Tomography Score (DWI-ASPECTS) and collateral status according to brush-sign presence.

Supplementary Table 1. Factors associated with diffusion-weighted imaging–Alberta Stroke Program Early Computed Tomography Score (DWI-ASPECTS) <7 in univariable and multivariable analyses for M1 MCA segment occlusion patients

	**crude OR (95% CI)**	**p-value**	**adjusted OR (95% CI)[a]**	**p-value**
Age	0.99 (0.97-1.00)	0.06	-	
Male	2.54 (1.58-4.12)	**<0.01**	2.11 (1.16-3.87)	**0.01**
NIHSS score	1.15 (1.10-1.20)	**<0.01**	1.15 (1.08-1.22)	**<0.01**
Brush-sign	1.74 (1.07-2.82)	**0.02**	-	-
Poor collateral status	15.43 (8.86-27.62)	**<0.01**	12.74 (7.02-23.93)	**<0.01**
Tandem occlusion	1.71 (0.43-6.1)	0.42	-	-

OR: odds ratio, CI: confidence interval, NIHSS: national institute of health stroke score, MCA: middle cerebral artery, ICA: internal carotid artery

[a] Model was adjusted for age, sex, NIHSS score and collateral status (brush-sign and tandem occlusion not retained by the backward selection)
