# Supplementary figures and images for: Does the Brush-Sign Reflect Collateral Status and DWI-ASPECTS in Large Vessel Occlusion?
Source: Front Neurol. 2022 Mar 2;13:828256. doi: 10.3389/fneur.2022.828256 (PMC8924293; doi:10.3389/fneur.2022.828256)

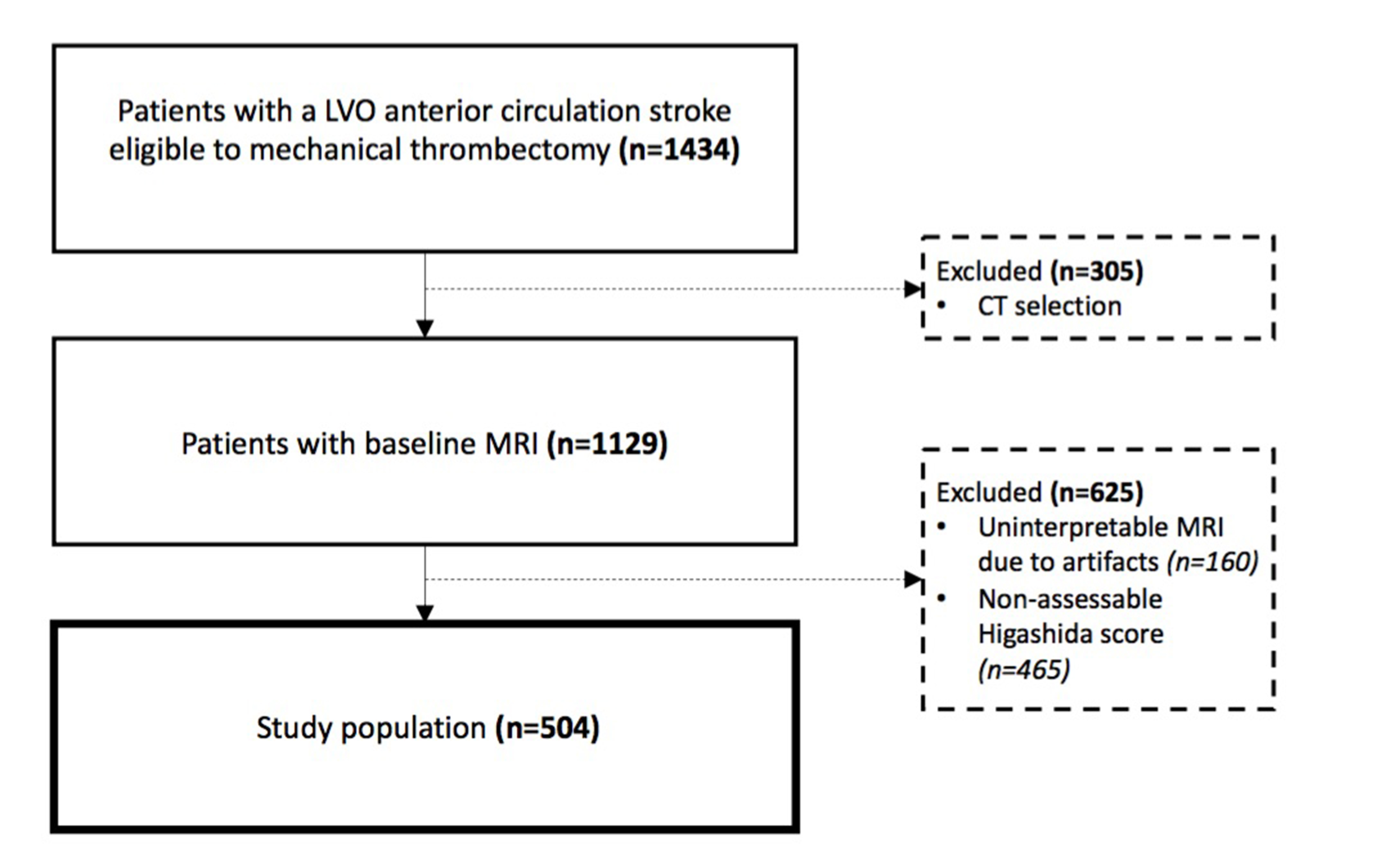

Supplement: Supplementary file 2 [file Image_1.JPG]

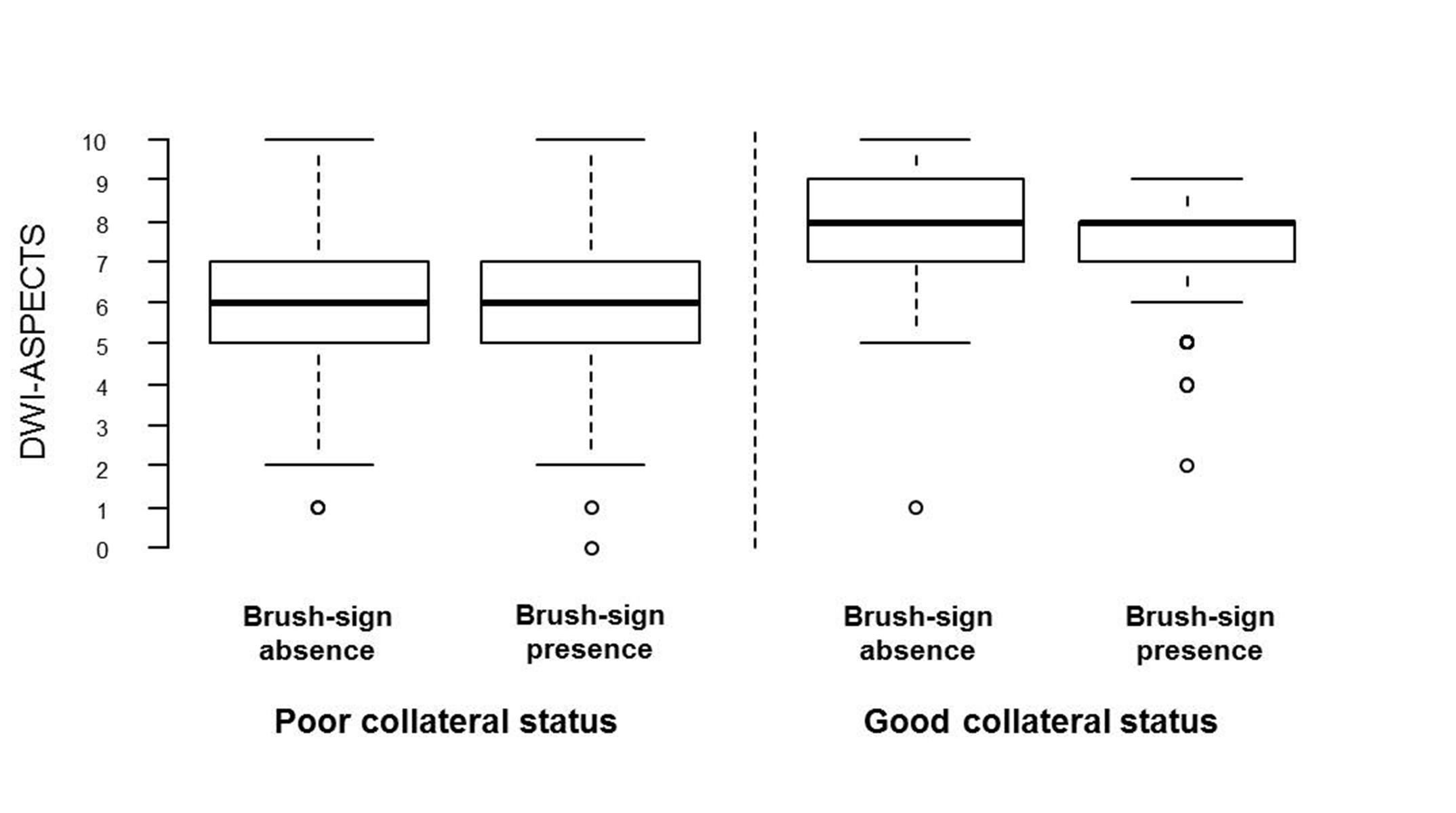

Supplement: Supplementary file 3 [file Image_2.JPG]
